# Sensitivity of Nitrogen K-Edge X-ray Absorption to Halide Substitution and Thermal Fluctuations in Methylammonium Lead-Halide Perovskites

**DOI:** 10.1021/acs.jpcc.1c02017

**Published:** 2021-04-09

**Authors:** Cody M. Sterling, Chinnathambi Kamal, Gabriel J. Man, Pabitra K. Nayak, Konstantin A. Simonov, Sebastian Svanström, Alberto García-Fernández, Thomas Huthwelker, Ute B. Cappel, Sergei M. Butorin, Håkan Rensmo, Michael Odelius

**Affiliations:** † Department of Physics, Stockholm University, AlbaNova University Center, SE-106 91 Stockholm, Sweden; ‡ Theory and Simulations Laboratory, HRDS, Raja Ramanna Centre for Advanced Technology, 452013 Indore, India; § Department of Physics and Astronomy, Uppsala University, Box 516, SE-751 20 Uppsala, Sweden; ∥ TIFR Centre for Interdisciplinary Sciences, Tata Institute of Fundamental Research, 36/P, Gopanpally Village, Serilingampally Mandal, 500046 Hyderabad, India; ⊥ Division of Applied Physical Chemistry, Department of Chemistry, KTH Royal Institute of Technology, SE-100 44 Stockholm, Sweden; # Swiss Light Source, Paul Scherrer Institut, WLGA/212, Forschungsstrasse 111, 5232 Villigen, Switzerland

## Abstract

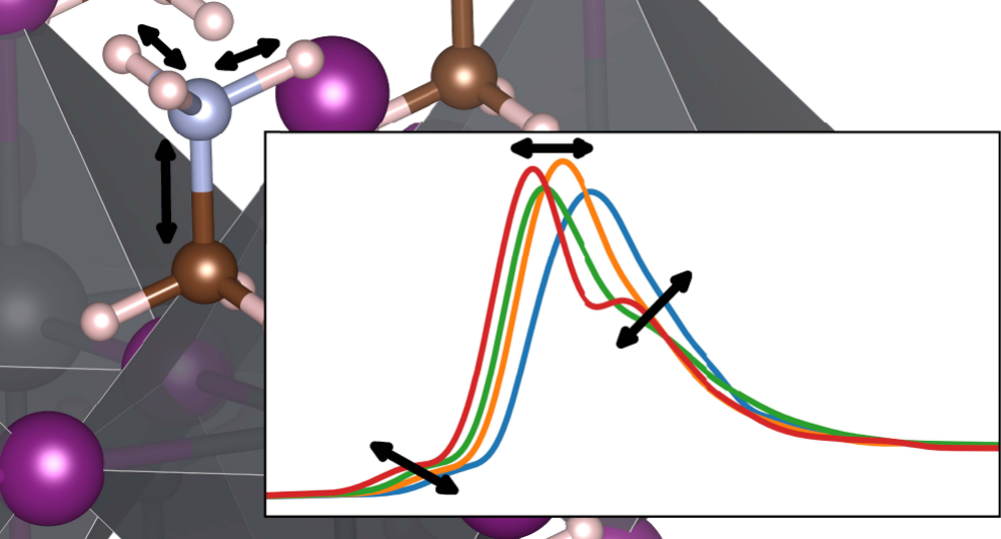

The performance of hybrid perovskite materials in solar cells crucially depends on their electronic properties, and it is important to investigate contributions to the total electronic structure from specific components in the material. In a combined theoretical and experimental study of CH_3_NH_3_PbI_3_—methylammonium lead triiodide (MAPI)—and its bromide cousin CH_3_NH_3_PbBr_3_ (MAPB), we analyze nitrogen K-edge (N 1s-to-2p*) X-ray absorption (XA) spectra measured in MAPI and MAPB single crystals. This permits comparison of spectral features to the local character of unoccupied molecular orbitals on the CH_3_NH_3_^+^ (MA^+^) counterions and allows us to investigate how thermal fluctuations, hydrogen bonding, and halide-ion substitution influence the XA spectra as a measure of the local electronic structure. In agreement with the experiment, the simulated spectra for MAPI and MAPB show close similarity, except that the MAPB spectral features are blue-shifted by +0.31 eV. The shift is shown to arise from the intrinsic difference in the electronic structure of the two halide atoms rather than from structural differences between the materials. In addition, from the spectral sampling analysis of molecular dynamics simulations, clear correlations between geometric descriptors (N–C, N–H, and H···I/Br distances) and spectral features are identified and used to explain the spectral shapes.

## Introduction

Halide perovskite materials, especially hybrid organic–inorganic perovskites, have in recent years been the focus of intense research as solar cells. Perovskites have the general formula ABX_3_, where the counterion A^+^ lies within the lattice of BX_3_^–^. Hybrid perovskites contain an organic counterion (A^+^) in cuboctahedral cages in the inorganic metal-halide lattice; the prototypical example is CH_3_NH_3_PbI_3_, methylammonium lead triiodide (MAPI), which has been extensively studied.^1−3^ Closely related systems with different counterions or halides are also studied.^4−6^ This class of materials has been shown to have excellent optoelectronic properties^7^ and reach a power conversion efficiency of over 25%,^8^ but many key questions remain open. For example, it is unclear to what extent the A^+^ counterion is important in the optoelectronic response and subsequent processes, or specifically, how much the lattice (construction and physical dynamics) of the material can change the electronic properties.^9^

In particular, previous work by Lindblad et al. suggests that an increase of electron-binding energy in the valence band of CH_3_NH_3_PbBr_3_ (MAPB) compared to MAPI is due to electronic properties of the two halides as opposed to geometric factors.^4^ This suggests that the choice of halide is an important factor when designing a solar cell from this class of materials. An investigation by Wang et al. shows how the band gap of mixed-halide MAPB/I and MAPB/CH_3_NH_3_PbCl_3_ can be tuned by adjusting the ratio of the two halides.^10^ These studies point toward important changes in the electronic structure when varying the halides in perovskites.

Others have investigated the geometric effects on the systems. Svane et al. investigated formate-based hybrid zinc perovskites with differing numbers of hydrogen bond donation sites in the formate counterion, demonstrating a positive correlation between the number of donating sites and the phase transition temperature.^11^ Herz and co-workers have investigated how lattice dynamics influences the electronic properties of pure metal-halide perovskites, indicating how lighter metals in the lattice lead the system to have lower exciton binding energies.^9^

X-ray absorption (XA) spectroscopy is a powerful probe of unoccupied orbitals in a system, and tuning the excitation energy in XA spectroscopy (XAS) to different core levels allows for an element specific analysis of complex materials. At the K-edge, XA spectra can often be readily assigned in a one electron picture, if the influence of core-hole relaxation is taken into account. Furthermore, since the 1s core levels are not influenced by spin–orbit coupling, relativistic effects have a small effect on the spectral shape for systems involving low-Z elements. The spectral features are associated with core-excited states, which in electronic structure calculations can be assigned to transitions from a 1s core level into unoccupied molecular orbitals with dipole transition probability related to the local p character of the orbitals. Because of the spatial extent of the unoccupied orbitals, the core-excited states are strongly influenced by coordination and chemical bonding. XA spectroscopy is a very sensitive chemical probe. In analogy to our measurements on the MA^+^ ion, previous work on ammonia and ammonium in aqueous solution indicates an effect of hydrogen bonding on the pre-edge features and overall spectral broadening to explain differences in the ammonia and ammonium N 1s XA spectra in water.^12^ Analogous studies of a series of ethylammonium with Et_*y*_NH_4–*y*_^+^ (*y* = 0...4; Et = C_2_H_5_) in solution is of direct relevance for comparison to our present data.^13^ Additionally, recently, Kot et al. showed that nitrogen does cause a noticeable change in the electronic structure of the MAPI system using X-ray photoelectron spectroscopy (XPS) and XAS on the N 1s edge.^3^

Complementary to the previous work of Lindblad et al. which primarily investigated the effects of halide substitution on occupied valence states^4^ and the work of Drisdell et al. on geometric effects in MAPI and MAPB on the Pb L_3_ absorption edge,^14^ in this paper, we examine unoccupied states with experimental N 1s XA spectra for MAPB and MAPI single crystals. The experimental spectra are assigned by comparison to computed spectra sampled from *ab initio* molecular dynamics (AIMD) trajectories. The sampled theoretical spectra are analyzed for correlations with several geometric descriptions by sorting individual spectra along specific geometric coordinates, namely, bond lengths of covalent and hydrogen bonds, and the spectral response and assignments are presented and discussed. A main motivation for this work is to obtain a more fundamental understanding of these changes by studying N 1s XA spectra from a geometric point of view, that is, how spectral changes can be explained in terms of geometric coordinates such as interatomic distances and asymmetries. We also aim to determine and understand the spectral differences between MAPI and MAPB.

## Methods

### Crystal Growth

Single-crystal cuboids of MAPB and MAPI with edge lengths of approximately 0.5 cm were obtained by a slow evaporation method. For MAPB, a 1.0 molar precursor solution was prepared by dissolving lead bromide and methylammonium bromide (1:1) in *N*,*N*-dimethylformamide. For MAPI, a 1.0 molar precursor solution was prepared by dissolving lead iodide and methylammonium iodide (1:1) in γ-butyrolactone. The resulting solutions were filtered with a 0.45 μm filter. Small single crystals began to grow at the bottom of the glass vials containing the solutions after slow evaporation of the solvent at 80 °C for MAPB and at 100 °C for MAPI. Seed crystals were collected and carefully placed in new glass vials containing filtered solution and were then allowed to grow into larger perovskite crystals. This step was repeated until crystals of the desired size (0.5 cm) were obtained; in this case, it took three times.

### Experimental XA Spectra

For the total electron yield (TEY) XA measurements, the crystals were epoxy-bonded to the sample plates and blade-cleaved in ultrahigh vacuum (UHV). The cleanliness of the cleaved crystal surfaces was checked using XPS with an excitation energy of 535 eV, making the measurements highly surface sensitive with an estimated probing depth between 2 nm for the N 1s core level and 5 nm for the valence band and XAS.^15,16^ The C 1s spectra showed no feature at energies expected for adventitious carbon from organic contamination but do show a feature from the methylammonium cation. Furthermore, the N 1s, Pb 4f, Br 3d, and I 4d spectra show only features expected from the perovskite; see Figure S1. The XAS measurements were performed on fresh spots, and the duration of the measurement was less than 30 min. No effect of the beam is expected in the spectra as we previously measured similar samples at the LowDosePES endstation for 7 h without any measurable change.^17^ TEY nitrogen 1s-to-2p* (N K-edge) XA measurements were performed at the synchrotron BESSY, at the soft X-ray beamline PM4, and at the LowDosePES endstation. The XAS measurements were performed with a photon bandwidth/resolution 125 meV (at the N K-edge) at room temperature in a UHV chamber with a base pressure of 1 × 10^–9^ mbar. Photon energy calibration was performed by measuring the kinetic energies of Au 4f photoelectrons excited by first- and second-order light and taking the difference, both below and above the absorption edge. Finally, a straight line background was subtracted.

### Molecular Dynamics

AIMD simulations for supercells of MAPI and MAPB in periodic boundary conditions were performed in the CP2K code^18^ under the same conditions with different starting supercell structures, as explained below. NPT simulations at 300 K and 0 atm were performed for 50 ps with a 0.5 fs timestep using orthorhombic simulation cells with fixed cell side ratios. Temperature was maintained using a four-chain Nosé-Hoover thermostat^19^ with a coupling time of 20 fs. The barostat time constant was also 20 fs and used a temperature tolerance of 100 K.

Forces for the dynamics were obtained using density functional theory (DFT)-based electronic structure calculations, with the PBE^20^ exchange–correlation functional and Grimme’s D3 van der Waals correction.^21,22^ The sampling of the electronic wave function was restricted to the Γ Brillouin zone point. The Gaussian and plane wave (GPW) method^23^ was used with a multigrid consisting of five grids and a cutoff of 600 Rydberg in combination with Goedecker–Teter–Hutter (GTH) pseudopotentials.^24,25^ Corresponding basis sets used were TZVP-MOLOPT-GTH for C, N, and H and DZVP-MOLOPT-SR-GTH for Pb and I/Br.^24,26^

**Figure 1:**
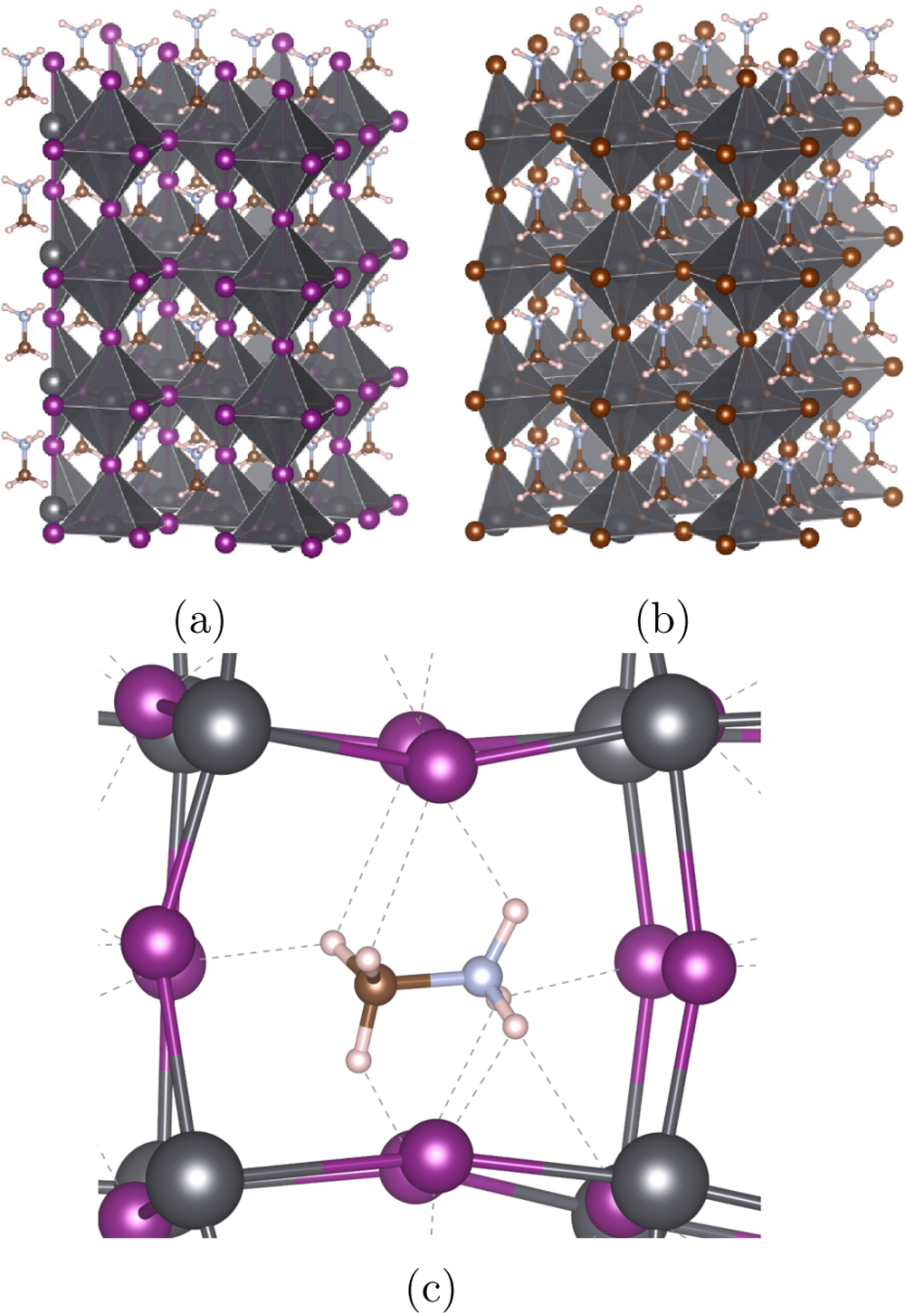
Starting supercell geometries for (a) 2 × 2 × 2 tetragonal β-MAPI and (b) 3 × 3 × 4 cubic α-MAPB, both with MA^+^ counterions aligned along the *z* axis. (c) shows a local orientation of the MA^+^ ion in MAPI during the course of the molecular dynamics simulation, with distances between the hydrogens and iodines shown with dashed lines. In (a–c), carbons are dark brown, nitrogens are blue, hydrogen is white, and lead is gray. Iodine is purple in (a,c), and bromine is light brown in (b).

Initial cell parameters were taken from Poglitsch and Weber’s study on various perovskite compositions and phases.^27^ For MAPI, we generated a 2 × 2 × 2 supercell from the unit cell of the tetragonal β phase (*a* = *b* = 8.855 Å; *c* = 12.659 Å). For MAPB, a supercell of comparable size (3 × 3 × 4) was generated from the unit cell of the cubic α phase (*a* = *b* = *c* = 5.901 Å). The MA^+^ ions were centered in the cavities of the inorganic PbI_3_ lattice, with the N–C bond aligned along the *z* axis. The starting geometries for MAPI and MAPB are shown in Figure 1.

### Computational XA Spectra

Snapshots of the cell geometries were sampled for five configurations at regular intervals during the molecular dynamics simulations. N 1s XA spectra, obtained within the half core-hole transition potential (TP_HH) approximation, were calculated for each nitrogen present in the supercell, yielding an overall sampling of 160 spectra for MAPI and 180 for MAPB. Inner-shell spectroscopies are enabled by all-electron calculations in the framework of the Gaussian augmented plane wave method,^28^ and we used the same DFT functional and settings as in the GPW-based AIMD simulation, except that a full potential description and all-electron basis sets were used for C, N, and H (6-311++G2d2p), whereas the TZVP-MOLOPT-SR-GTH pseudopotential basis sets and pseudopotential description were retained for Pb and I/Br. Gaussian convolution on the discrete transitions was performed using the normalized Gaussian curve with σ = 0.2 eV (full width at half-maximum as ). Due to limitations of the XA spectrum simulations, an ad-hoc shift of −2.86 eV was added to the calculated MAPI and MAPB spectra for direct comparison to the experiment.

## Results and Discussion

**Figure 2:**
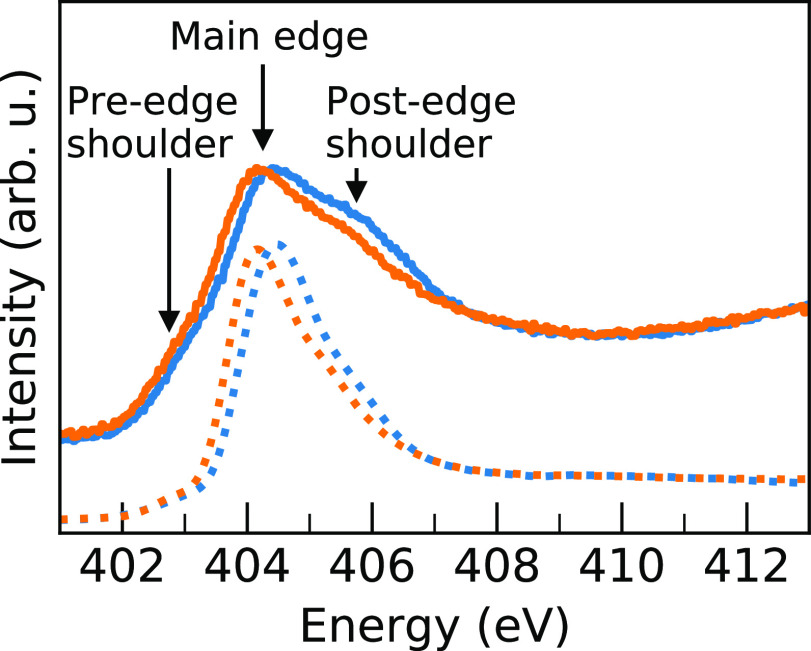
Comparison of experimental N K-edge XA spectra with the TEY for MAPB (solid blue) and MAPI (solid orange) with the corresponding calculated spectra for MAPB (blue dash) and MAPI (orange dash) shifted by −2.86 eV. The arrows indicate the pre-edge shoulder, the main edge, and the postedge shoulder.

Figure 2 shows the comparison between the experimental nitrogen K-edge XA spectra of MAPB and MAPI and theoretical spectra sampled over AIMD simulations of the supercell models depicted in Figure 1. As a first approximation, the experimental spectra look rather similar to each other with a maximum at about 404.3 eV and a similar shape. However, a closer inspection indicates that the MAPI spectrum is shifted by −0.23 eV in comparison to the MAPB spectrum, and it has a less pronounced shoulder at about 406 eV. Moreover, we note that the resonance at 415 eV seen in the extended range of the spectra belongs to the Pb N_5_ edge (Pb 4d-to-6p*; see Figures S2 and S3) and that the spectra also indicate some differences here. The shape of the experimental K-edge of MAPI resembles previous measurements.^15^ The primary aim of the simulations is to rationalize the differences in the N K-edge main edge positions of MAPI and MAPB and assign their general features.

The theoretical N 1s XA spectra for MAPI and MAPB in Figure 2 are averaged over all nitrogens among all sampled geometries. The two calculated spectra show the same characteristics: a pre-edge shoulder, a main edge absorption peak, and a postedge shoulder. The peak maxima of the theoretical spectra were aligned to the experimental MAPI spectrum, and the MAPB spectrum was shifted by the same energy. Because the calculated spectra do not reproduce the absolute excitation energies, we refer instead to the *relative* MAPI/MAPB shift which remains unchanged by shifting. The shift is performed to make the direct comparison between the calculated and experimental spectra more straightforward in the figure and is aligned to MAPI because those are the figures shown in the main body of this work. The maximum of the main peak intensity between MAPI and MAPB for the theoretical spectra is shifted by 0.31 eV, in good agreement with the experiment. Further shifting of the calculated spectra relative to each other such that their peak intensities align shows that they largely overlap in shape, indicating a nearly systematic shift between the two systems; this is shown in Figure S4. However, for the calculated MAPB spectrum, we observe a small increase in the postedge shoulder, 2 eV above the peak maxima, in agreement with the experimental findings.

Although the difference of the main peak intensity in the experimental and calculated XA spectra of MAPI and MAPB is well reproduced, the calculated spectra are much too narrow and the shapes and differences in the postedge region are not captured. This can be improved with a larger broadening, but consequently, the spectral detail (in particular the pre-edge shoulder) is reduced. The performance of the transition potential approximation has been evaluated before in N 1s XA spectra of ammonium species^12,13^ and in which similar artifacts related to the transition potential approximation have been observed. Thus, we probe the sensitivity in the calculated spectra to geometric variation only around the pre- and main-edge features.

**Figure 3:**
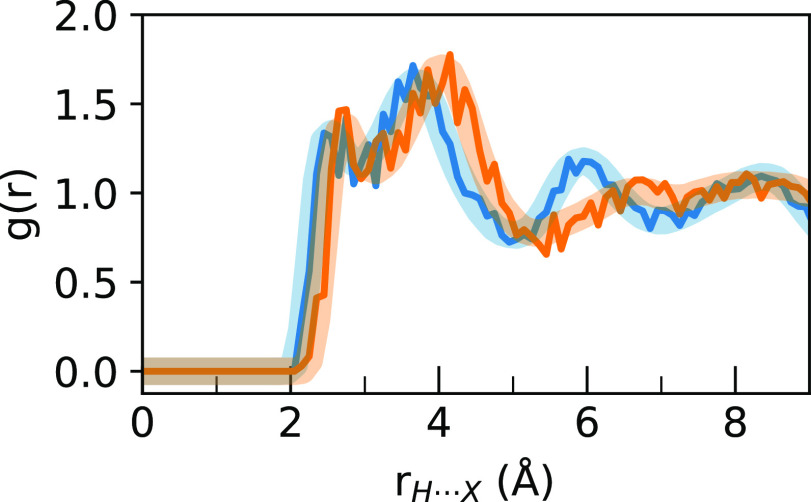
RDF of H···I (orange) and H···Br (blue) distances for nitrogen-attached hydrogens during the full simulation (transparent lines) and the sampled geometries (solid lines). For both systems, the good agreement validates the sampling as representative of the whole simulation.

To show that the sampling of configurations selected for spectrum geometries for MAPI and MAPB are representative of the AIMD trajectory, we compare the radial distribution functions (RDFs) of the sampled geometries and the whole simulation. A plot of this is shown in Figure 3 for the H···X distance, and the close agreement between the MAPI and MAPB RDFs demonstrates that the five sampled geometries provide a good representation of both systems.

**Figure 4:**
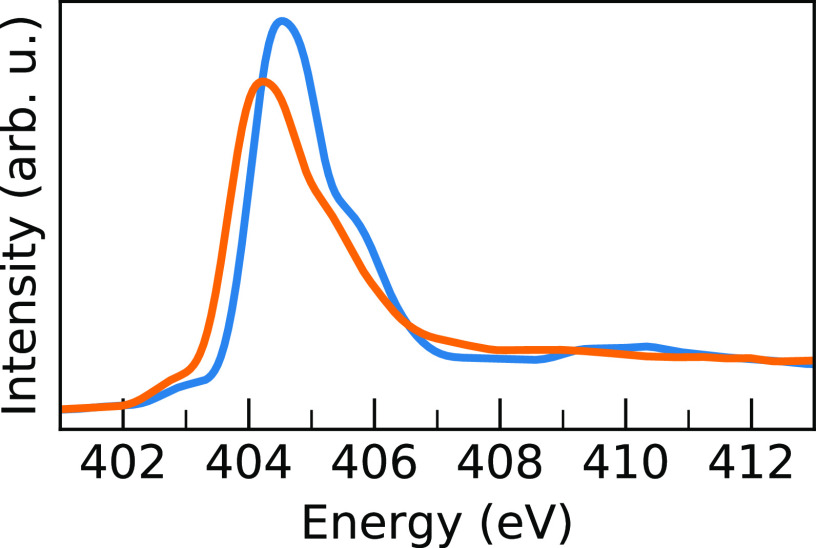
Comparison of N K-edge spectra for a MAPI configuration (orange) and the same configuration with I replaced by Br (blue). Peak maxima occur at 404.23 and 404.52 eV. This peak-intensity shift of 0.29 eV matches the shift from Figure 2 of 0.31 eV, indicating that the shift is not mainly due to the cell structure.

However, due to the systems having different geometries and different crystal structures (tetragonal MAPI vs cubic MAPB), it is unclear whether the −0.3 eV spectral shift, observed in Figure 2, is due to a chemical difference between iodine and bromine or the geometric differences of the systems. To investigate the sensitivity to the structure, we took one configuration of MAPI and replaced all iodines with bromines to create an identical tetragonal MAPB and recalculated the XA spectrum. The result of this is shown in Figure 4. The resulting MAPI to MAPB peak shift at a common geometry is 0.29 eV, essentially the same as the shift for the aggregate sampled spectra. The MAPB spectrum also has a slightly more prominent postedge shoulder than in Figure 2, but this is not unusual compared to some of the other individual spectra. We notice that the artificially long distances around Br seem to influence the intensity of the spectrum. However, this clearly indicates that the peak intensity shift is not due to differences in the geometries of the systems but rather an electronic difference between the halides. We also notice that the differences in band gap between the two systems—1.55 eV for MAPI^29,30^ and 2.28 eV for MAPB,^10^ a difference of 0.73 eV—is twice the shift in the absorption energies. An additional comparison is done between the PBE and BLYP functionals (Figure S5) to investigate the functional effects, but these functionals showed similar MAPI–MAPB shifts and spectral features.

The experimental spectrum is an average of many different nitrogens in various accessible local environments; therefore, in order to effectively compare theoretical results to it, we similarly need to simulate XA spectra for a range of nitrogens in different local environments. Each nitrogen site in each sampled AIMD configuration requires a separate N 1s XA spectrum calculation. A total of 160 individual N spectra were calculated for MAPI and 180 for MAPB. Averaged together, these spectra are then meaningfully compared to experimental results, as shown in Figure 2.

However, instead of simply averaging all spectra together, we can organize the spectra into “sub-averages” according to some criterion to see how that affects the spectral shape and features. This can be done by choosing some metric (e.g., N–C distance), ordering the spectra accordingly, and averaging them into blocks for a fixed distance range. The averaging aims to smooth out any other effects to isolate only the effect of the coordinate we are interested in under the assumption that they are not strongly correlated. This averaging procedure is, however, vulnerable to situations where a block may have only one or two spectra in it. These blocks are not well-averaged and therefore cannot properly be compared to the other well-averaged blocks in terms of trends along the coordinate, and thus they are not shown in the block average comparison figures.

**Figure 5:**
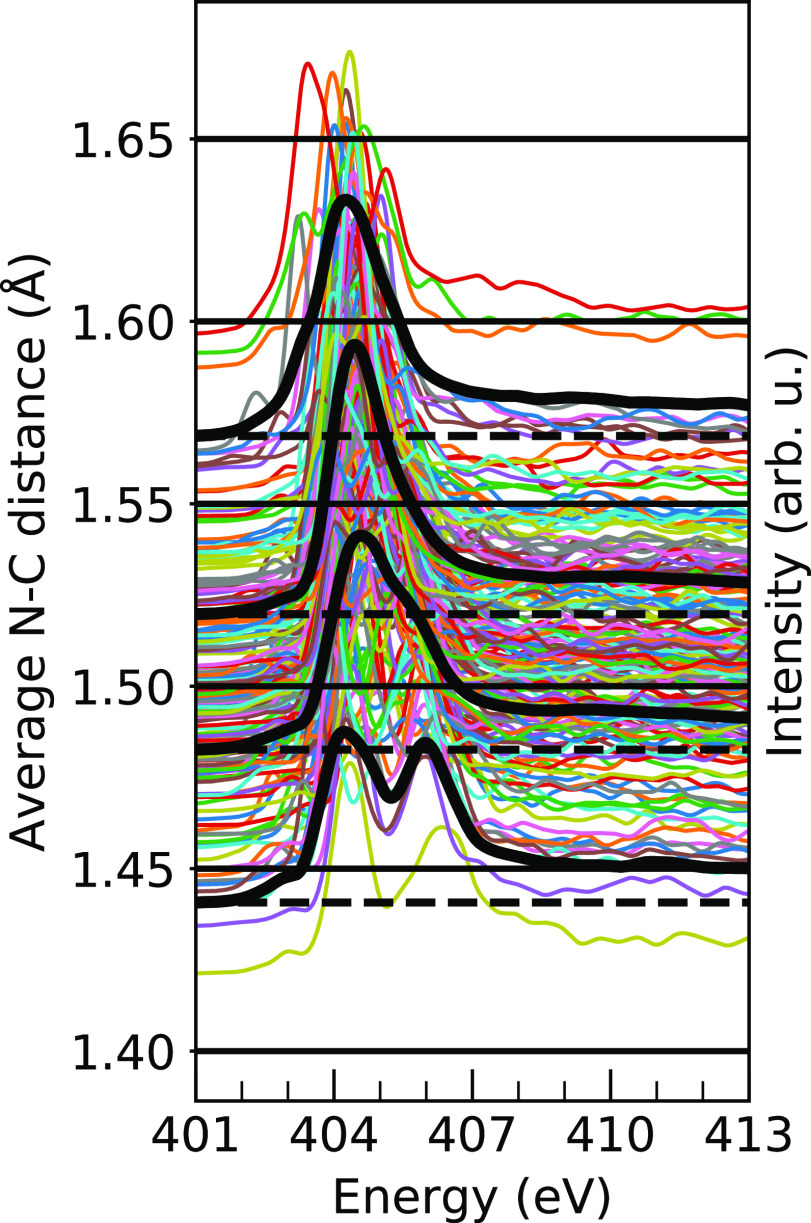
All individual N 1s XA spectra for the sampled MAPB geometries (thin lines) sorted by the N–C distance for each respective nitrogen. The 0.05 Å blocks are delineated with horizontal black lines, and the block spectra averages are shown as thick black lines. The block averages are reproduced in Figure S6.

The distance block averaging is illustrated graphically in Figure 5 for the N–C distance in MAPB. For each nitrogen, the spectrum is plotted as a thin line and offset vertically according to its N–C distance, as shown on the left axis. These are then split into 0.05 Å blocks, and all spectra in each block are averaged, represented by the thick black spectral lines. It is these thick black spectral lines that provide the appropriate information of interest regarding the averaged effect of the coordinate on the spectra, so in the presentation below, we report only those averages, centered on the same starting point to better compare relative changes among the spectra.

**Figure 6:**
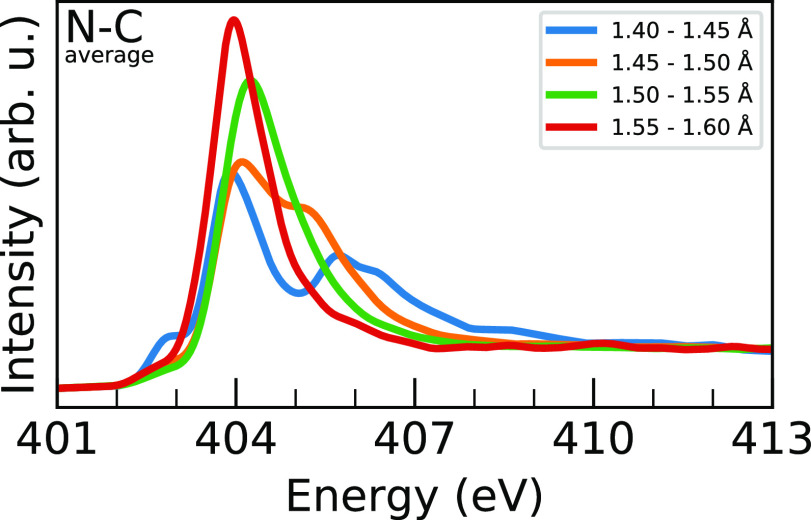
Block-averaged MAPI N 1s XA spectra ordered by N–C distance in 0.05 Å blocks from 1.40 to 1.60 Å. Explanation of the trends is given in the text, and the block-averaging procedure is given in Figure 5.

Since we are interested in geometric effects on the nitrogen XA spectrum, our focus is on coordinates that involve the MA^+^ molecular ion. First, we report the intramolecular coordinates, N–C distance, and average N–H distance. Starting with the N–C coordinate, as shown in Figures 6 and S6, a clear trend is seen for both MAPI and MAPB that a decrease in the N–C distance gives rise to a splitting of the main peak into two peaks; one which stays around 404 eV and the other which progressively blue shifts. The average sampled N–C bond lengths of MAPI and MAPB are both 1.50 Å, which explains the slight postedge shoulder seen in Figure 2 as a combination of orange and green lines. The dependence on the N–C distance is clearly shown, but it is not a degree of freedom changing between MAPI and MAPB.

**Figure 7:**
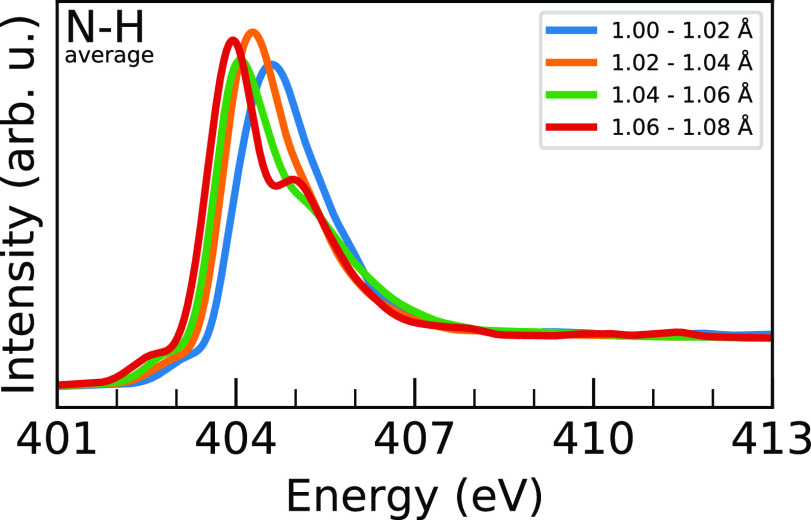
Block-averaged MAPI N 1s XA spectra ordered by the average N–H distance in 0.02 Å blocks from 1.00 to 1.08 Å. Explanation of the trends is given in the text, and the block-averaging procedure is given in Figure 5.

Next, we turn to analyzing the other pertinent intramolecular distance coordinate, the average N–H distance for the hydrogens attached to each nitrogen. These data are shown in Figures 7 and S7. One notable trend that these plots show is that the longer N–H distances seem to exhibit more of the postedge shoulder behavior associated with the short N–C distances shown previously. An analysis of the sampled structures does show a slight inverse relationship between these coordinates, as shown in Figure S8. Specific to just the N–H distances, however, the plots show that increasing N–H distance is associated with a red-shifting of the whole spectrum, particularly when examining the main peak positions. As with the N–C analysis, this is borne out in the combined average spectra. The average N–H distance for both systems is 1.04 Å, which is between the orange and green plots. For MAPI, these two peaks average to a maximum intensity at 404.18 eV, and for MAPB, they average to 404.49 eV, comparing favorably to the total averages of 404.2 and 404.47 eV, respectively. In analogy with previous studies of ethylamines,^13^ the trends in the distance dependence in Figures 6 and 7 can be understood in terms of shape resonances for the antibonding σ_N–C*_ and σ_N–H*_ orbitals, and the splitting of the XA spectrum into a main-edge and a separate postedge figure can be rationalized by the anticorrelation of the N–C and N–H distances observed in Figure S8.

**Figure 8:**
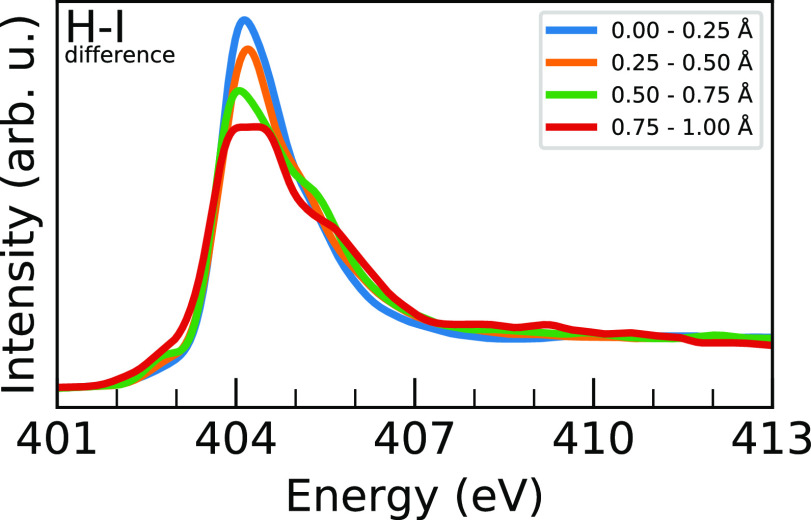
Block-averaged MAPI N 1s XA spectra ordered by the difference in the shortest and longest nitrogen H···I distance in 0.25 Å blocks from 0 to 1 Å. Explanation of the trends is given in the text, and the block-averaging procedure is given in Figure 5.

As stated in the introduction, based on previous work by Ekimova et al.,^12^ we believe hydrogen bonding between the nitrogen-attached hydrogens and the lattice I/Br to be important. Specifically, based on their results, it is expected that increasing N–H hydrogen bond donation will cause a broadening of the spectrum. Given that there is a hydrogen bonding effect on the spectrum, one naturally would expect to see some difference between a nitrogen with all hydrogens strongly donating and one with no hydrogens donating. However, it would also be expected to be possible to distinguish between symmetrically donating nitrogens and asymmetrically donating nitrogens. To investigate this, we order the spectra by the difference between the shortest and longest H···I or H···Br distances (Figures 8 and S9, respectively) for each nitrogen; a lower value indicates symmetry, while a higher value indicates asymmetry, though this does not necessarily give information about the strength or number of hydrogen-bonding interactions. The results of this analysis indicate that there is a clear effect of this asymmetry on the peak intensity and broadening of the spectrum. Additionally, in contrast to previous coordinates, this asymmetry also correlates with the strength of the pre-edge shoulder. This is also in line with the previous results of Ekimova et al., who argued that the appearance of an ammonia pre-edge was due to differences in the symmetry of the lowest unoccupied molecular orbital (LUMO) as compared to the symmetric ammonium which had no pre-edge and that the slight pre-edge for aqueous ammonium is related to instantaneous distortions of symmetry.^13^

To complement the block-averaging analysis and gain a deeper understanding of the correlation between the spectra and investigated geometric coordinates, we also carried out a detailed analysis of molecular orbitals involved in the prominent transitions and the corresponding unoccupied partial density of states (PDOS). For this purpose, we have chosen two specific configurations corresponding to methylammonium in MAPI with relatively shorter (1.475 Å) and longer (1.497 Å) N–C bond distances. In the former configuration, there is also a large pre-edge intensity associated with one very long nitrogen H···I distance and a large asymmetry (H_1_···I = 3.48 Å, H_2_···I = 2.82 Å, and H_3_···I = 2.65 Å; asymmetry = 0.83 Å), whereas in the other configuration with an equally large asymmetry, there is instead one very short nitrogen H···I distance (H_1_···I = 3.17 Å, H_2_···I = 2.91 Å, and H_3_···I = 2.37 Å; asymmetry = 0.80 Å). Therefore, we have selected configurations that allow us to investigate the mechanisms for spectral response both to changes in the N–C bond distance, as presented in Figure 6, and to variations in hydrogen bonding, as presented in Figures 7 and 8.

**Figure 9:**
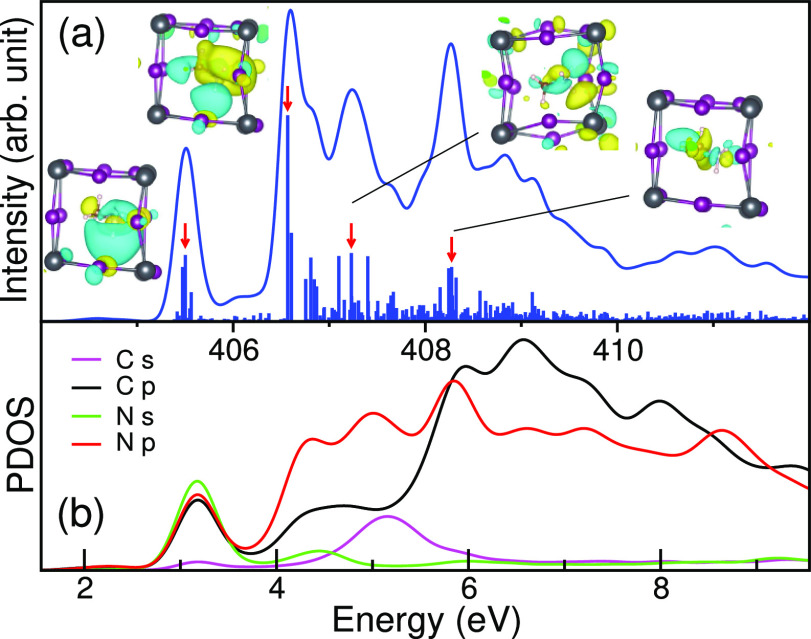
(a) Individual N 1s XA spectrum (line) for a specific N atom in MAPI with a shorter N–C distance. The molecular orbitals (bars) around the MA^+^ ion are given, and the specific ones corresponding to the inlaid images are indicated by red arrows. (b) Corresponding unoccupied PDOS for the system.

**Figure 10:**
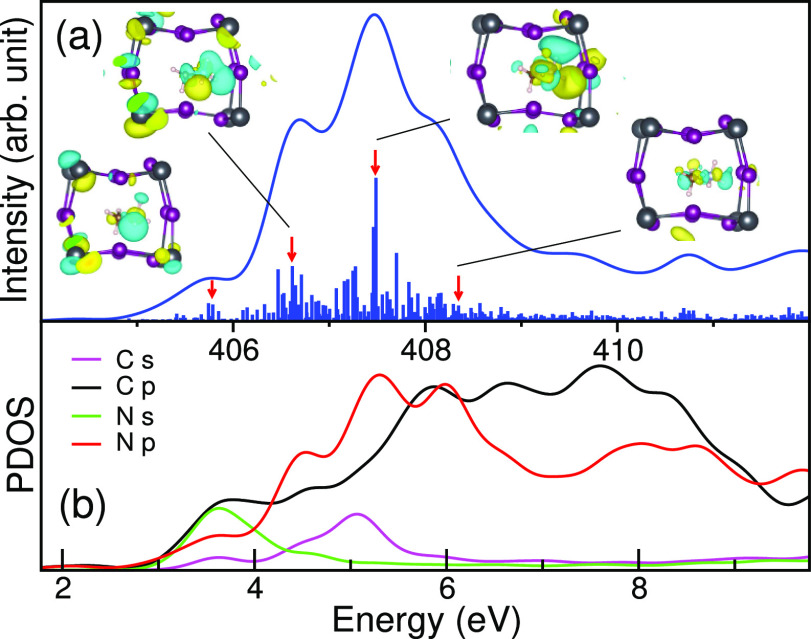
a) Individual N 1s XA spectrum (line) for a specific N atom in MAPI with a longer N–C distance. The molecular orbitals (bars) around the MA^+^ ion are given, and the specific ones corresponding to the inlaid images are indicated by red arrows. (b) Corresponding unoccupied PDOS for the system.

The results of the orbital analysis are presented in Figures 9 and 10. These figures also contain molecular orbitals around MA^+^ from the strong transitions in pre-, main-, and postedge regions in the TP_HH calculations and corresponding PDOS of carbon and nitrogen in the core-excited MA^+^ ion. For reference, the ground-state Kohn–Sham orbitals of an isolated MA^+^ ion are given in Figure S10. It should be noted that the *a*_1_ and *e* symmetries correspond to the σ and π character along the N–C bond.

We note that the intensity of spectra readily follows the PDOS of N p orbitals, but the XA intensity is more pronounced near the edge where the N 2p contribution dominates. The results of the PDOS in Figures 9 and 10 clearly indicate that the states in the pre-edge region for both cases have major contributions from N s, N p, and C p orbitals. They possess the character of the LUMO, with 6*a*_1_ symmetry, of the isolated MA^+^ ion in Figure S10. However, depending on the hydrogen bond environment, the cyan orbital lobe around nitrogen has been strongly distorted due to the interaction of the MA^+^ ion with the surrounding Pb–I cage.

For the configuration in Figure 9, there is sufficient room for a large lobe of the 6*a*_1_ orbital to form, whereas in Figure 10, the strong confinement around the ammonium group results in a significant quenching of the orbital amplitude. Due to this, the N p character in the states at the pre-edge region has been significantly reduced as seen in the PDOS. In both cases, we observe a localization of the 6*a*_1_ lobe in the direction of the longest H···I hydrogen bond and a hybridization with a small amount of I 5p to form antibonding σ*(H···I) hydrogen bonding character. As noted in the block averaging in Figure 8, the intensity in the pre-edge region is enhanced by asymmetry, which increases the N p character in the transition as seen in the N p PDOS, and here we note that predominantly long hydrogen bonds contribute to the pre-edge feature as observed in liquid water.^31,32^

The transitions identified as contributing to the second (406.6 eV) and third (407.2 eV) features have more mixed characters but resemble the LUMO + 1 (3*e*) and LUMO + 3 (4*e*) orbitals of the isolated MA^+^ ion, both having π(N–C) symmetry and antibonding σ*(N–H) character. The strong feature at 408.3 eV in the postedge region in Figure 9 is due to states which have significant contributions from antibonding σ*(N–C) character associated with orbital 8*a*_1_, pushed up in energy by the short N–C distance, and appears much less pronounced in Figure 10. The molecular states (7*a*_1_ and 9*a*_1_) with strong C s and C p_*z*_ (along N–C bond) characters have not contributed significantly to any prominent peaks. The analysis indicates that the main peak intensity corresponds to states with the π(N–C) symmetry and antibonding σ*(N–H) character but with contributions of states with σ*(N–C) orbital character for longer N–C distance, which is redistributed to the postedge peak at a reduced N–C distance.

In addition to the previously mentioned fact that the MAPI and MAPB simulations used different crystal structures at 300 K (tetragonal and cubic, respectively), the fact that bromine is a smaller atom than iodine means the inorganic lattice grid in the material is smaller, and that will affect any direct comparison of MA–lattice distances between the two systems. The RDFs for N–I/Br, H–Pb (for nitrogen hydrogens), and N–Pb are shown in the Supporting Information in Figures S11–S13. In short, these RDFs appear to show similar distributions for both systems when accounting for the difference in ion sizes (i.e., relative peak behavior).

**Figure 11:**
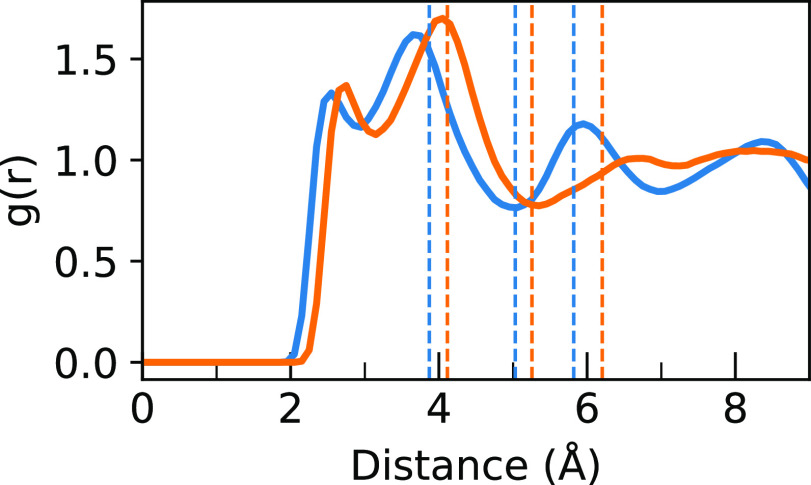
RDF of H···Br (blue) and H···I (orange) distances for nitrogen-attached hydrogens during the simulations of MAPB and MAPI. The distances with an integral of 4, 8, and 12, corresponding to fractions of the Pb–I/Br cage for each MA^+^ molecular ion, for each RDF are shown in their respective colors from left to right.

The H···I/Br (for nitrogen-attached hydrogens) RDFs, in contrast, show qualitatively different long-range behavior between the two systems, as seen in Figure 11. Vertical lines are drawn for each RDF corresponding to an integral value of 4, 8, and 12, from left to right, corresponding to fractions of the 12-atom Pb–I/Br cage that each MA^+^ molecular ion is in. For short distances (<5 Å), the RDFs look similar except for a slight expected lengthening effect for MAPI, but for long distances (>5 Å), it is seen that MAPB continues to have more distinct peaks as compared to MAPI which is much more smoothed out in its distribution.

**Figure 12:**
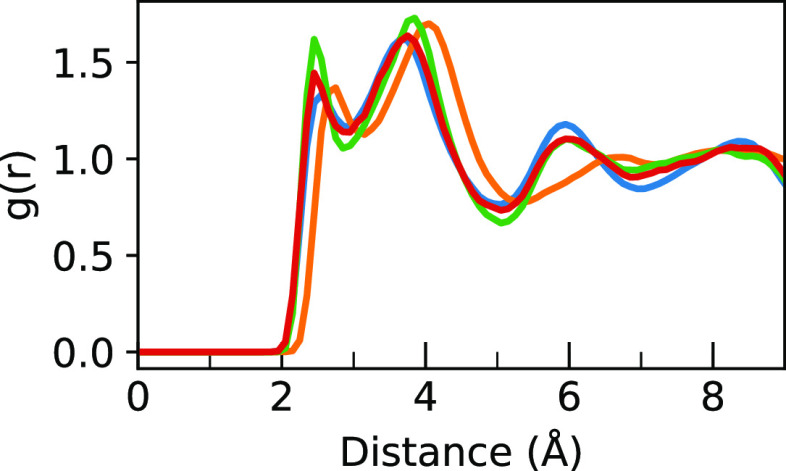
RDF of H···Br (blue) and H···I (orange) distances for nitrogen-attached hydrogens during the 50 ps simulations of cubic MAPB and tetragonal MAPI are compared to tetragonal MAPB RDFs after an extra 10 ps of simulation at 200 K (green) or 300 K (red). It can be seen that both tetragonal MAPB RDFs match the cubic MAPB RDF much more than the tetragonal MAPI RDF.

To determine if this effect was due to differences in the crystal structure or supercells, which are somewhat constrained in the simulation, we took the end of the MAPI simulation and replaced all iodines with bromines to make an identical tetragonal MAPB system. This system was rescaled to the experimental tetragonal β-MAPB parameters from Poglitsch and Weber^27^ and run in an NPT MD simulation as before for another 10 ps. Two separate simulations were performed, one at 300 K as before and another at 200 K because the tetragonal–cubic transition in MAPB occurs at 237 K. The RDFs for these short simulations were then compared to the previous RDFs, as shown in Figure 12. In this figure, it can be seen that there is almost no difference between the 200 and 300 K simulations and that both of these simulations match up very well with the cubic MAPB RDF. Furthermore, the tetragonal MAPB spectra at both 200 and 300 K (Figures S14 and S15, respectively) are very similar to the original cubic MAPB spectrum, not the tetragonal MAPI spectrum. This indicates that the long-range difference observed here is not due to crystal structure differences but instead due to the difference in atomic radii between the two halide ions.

To summarize the Results section, careful measurements of nitrogen K-edge XA spectra of MAPI and MAPB show distinct spectral differences which we have dissected theoretically by a combination of AIMD simulations and spectrum simulations on the two compounds. The combination allows us to determine aspects of the spectra related to structural changes and to purely electronic changes from halide substitution.

## Conclusions

In a combined experimental and theoretical study, we compared the N 1s XA spectra of room-temperature structures of tetragonal MAPI and cubic MAPB. The experimental data, coming from measurements on single crystals in UHV, allow for clean N 1s XA spectra largely free from beam damage and a detailed comparison of MAPI and MAPB. Theoretical models compare favorably with the experimental spectra. We observe an approximately 0.3 eV blue shift of the MAPB spectrum relative to the MAPI spectrum, which is not due to the crystal structure differences but due to the difference in band gaps, indicating that electronic structure information is encoded in N 1s XAS.

AIMD simulations were performed and sampled for the XA spectrum analysis. In order to gain a more detailed understanding of the N 1s XA spectra of hybrid perovskites, the individual nitrogen spectra were then ordered and averaged along various intra- and intermolecular coordinates relative to methylammonium counterion atoms—specifically the N–C bond length, average N–H length, and H···I/Br asymmetry—to determine the effects of these distances on the XA spectra. It was found that a shorter N–C length corresponds to the appearance of a higher-energy peak that leads to a postedge shoulder feature in the overall spectrum, and a shorter N–H length corresponds to blue-shifting the entire spectrum, which is also borne out in the overall calculated spectrum. It was also shown that the H···I/Br asymmetry corresponds to a decrease in the main peak intensity and the appearance of the pre-edge shoulder. These trends are generally clearer in the MAPI spectra but are also represented well in the MAPB spectra shown in the Supporting Information.

Finally, it is reported that the long-range behavior of the H···I/Br distances are different between MAPI and MAPB according to their RDFs. This effect holds even when accounting for the differing crystal structures by performing a tetragonal MAPB simulation, and we attribute it to the difference in atomic radii.

These results provide information on how the local structure of the material can be seen from its effects on the XA spectra. In particular, it points to the role of hydrogen bonding (asymmetry) in the spectrum, and thus the electronic structure, which can be important for further development of hybrid perovskite materials for solar cell applications.

## Supporting Information Available

Extra calculated XA spectrum comparison plots, the MAPB block-averaged plots, additional RDFs, isolated MA^+^ orbitals, and a comparison of experimental methods for the MAPI spectrum (PDF)
