# Supplementary material for: Sensitivity of Nitrogen K-Edge X-ray Absorption to Halide Substitution and Thermal Fluctuations in Methylammonium Lead-Halide Perovskites
Source: J Phys Chem C Nanomater Interfaces. 2021 Apr 9;125(15):8360–8. doi: 10.1021/acs.jpcc.1c02017 (PMC8162417; doi:10.1021/acs.jpcc.1c02017)
Supplement: Supplementary file 1 — jp1c02017_si_001.pdf [file jp1c02017_si_001.pdf]

Supporting Information:

Sensitivity of Nitrogen K-edge X-ray Absorption to Halide Substitution and Thermal Fluctuations in Methylammonium Lead Halide Perovskites

Cody M. Sterling,[†] Chinnathambi Kamal,[†,‡] Gabriel J. Man,[¶] Pabitra K. Nayak,[§] Konstantin A. Simonov,[¶,#] Sebastian Svanström,[¶] Alberto García-Fernández,[‖] Thomas Huthwelker,[⊥] Ute B. Cappel,[‖] Sergei M. Butorin,[¶] Håkan Rensmo,[¶] and Michael Odelius[*,†]

†*Department of Physics, Stockholm University, AlbaNova University Center, SE-106 91 Stockholm, Sweden*

‡*Theory and Simulations Laboratory, HRDS, Raja Ramanna Centre for Advanced Technology, Indore - 452013, India*

¶*Department of Physics and Astronomy, Uppsala University, Box 516, SE-751 20 Uppsala, Sweden*

§*TIFR Centre for Interdisciplinary Sciences, Tata Institute of Fundamental Research, 36/P, Gopanpally Village, Serilingampally Mandal, Hyderabad, 500046, India*

‖*Division of Applied Physical Chemistry, Department of Chemistry, KTH Royal Institute of Technology, SE-100 44 Stockholm, Sweden*

⊥*Swiss Light Source, Paul Scherrer Institut, WLGA/212, Forschungsstrasse 111, 5232 Villigen, Switzerland*

#*Present address: Department of Materials and Process Development, Swerim AB, Box 7047, Stockholm, Sweden*

E-mail: odelius@fysik.su.se

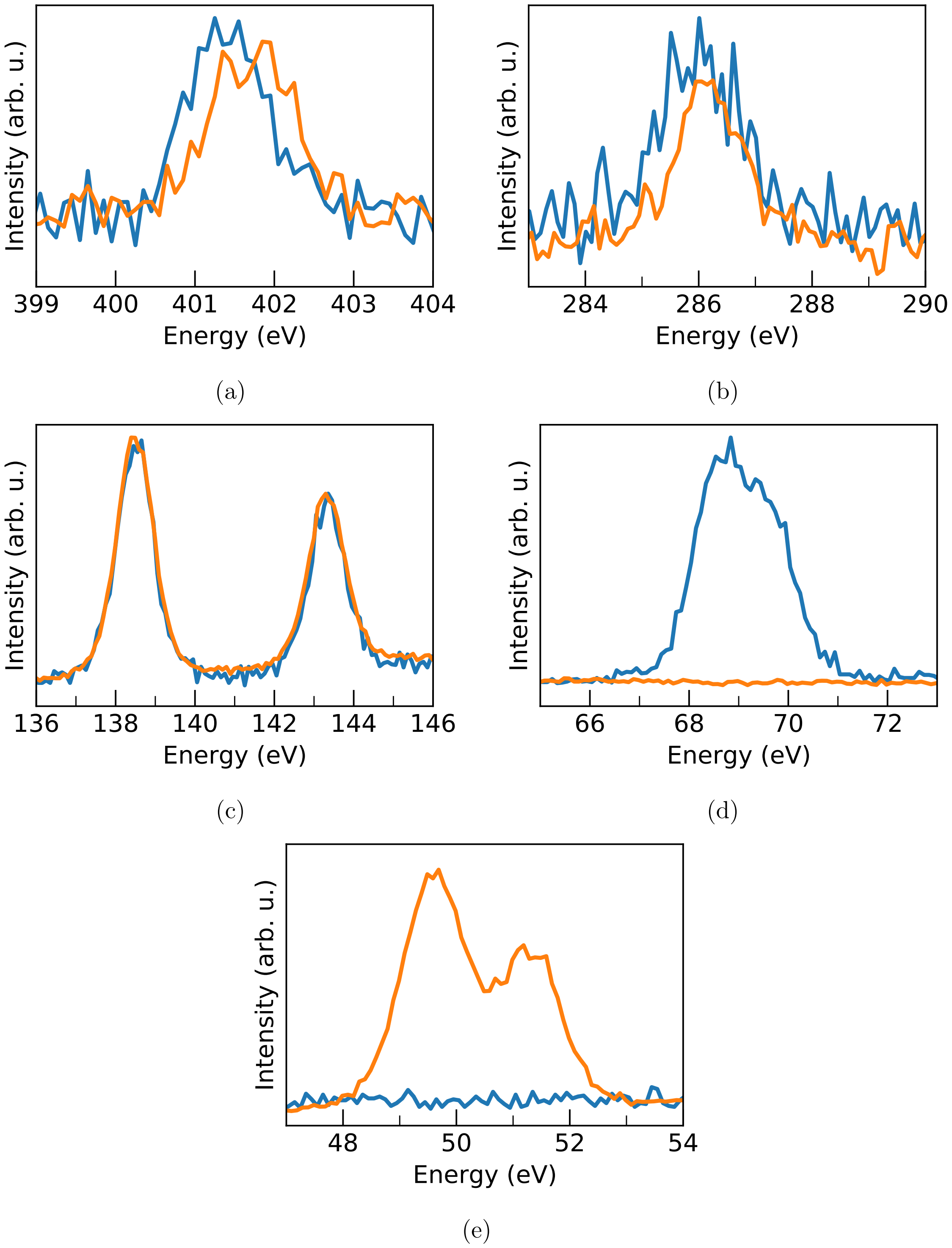

Figure S1: The XPS signal from a) N 1s, b) C 1s, c) Pb 4f, d) Br 3d, and e) I 4d core levels of $MAPbBr_3$ (blue) and $MAPbI_3$ (orange) measured using a photon energy of 535 eV. Intensity normalised and energy calibrated against Pb 4f at 138.5 eV.

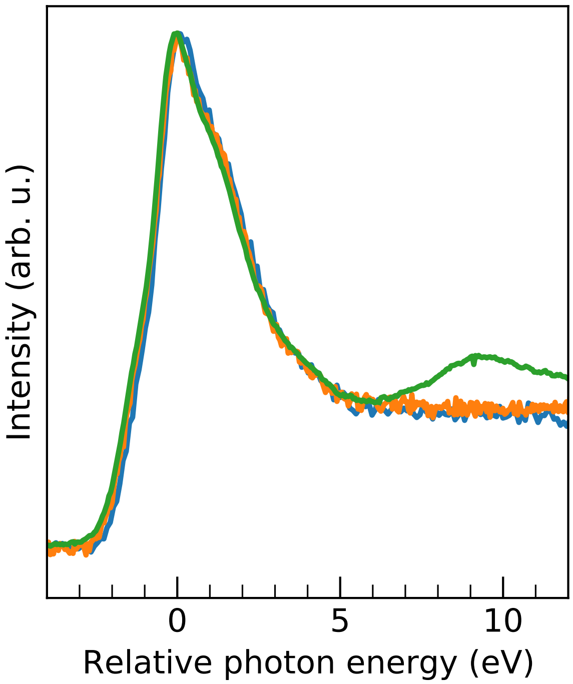

Figure S2: Three independent N K-edge XAS measurements recorded from MAPI single crystals. Measurements were shifted to place the main edge intensity at 0 eV. Blue: MAPI after subtraction of PbI_2 signal. Orange: MAPI partial electron yield. Green: MAPI total electron yield. Experimental details on the crystal synthesis are given in the text.

In Figure S2 we show three different spectra from measurements of N K-edge ($1s$-to-$2p^*$) X-ray absorption measurements of single crystals. The spectra are aligned to the MAPI total yield spectrum reported in the main manuscript (Figure 2, solid orange). This is done to show that independent measurements recorded from different crystals give similar results.

Also shown is the spectrum obtained after removal the signal recorded for the PbI_2 sample over the same region measured on a similar crystal (see below) measured using the same set-up at the PM4 beamline. This shows that the feature 10 eV above N K-edge in the MAPI, as well as the MAPB shown in the main manuscript, is linked to a lead absorption feature (Pb $4d$-to-$6p^*$).

Moreover, the feature 10 eV above the N K edge is also lacking in a partial electron yield spectrum measured at the FlexPES beamline at the synchrotron facility MAX-IV in Sweden. These measurements were performed in partial yield detection mode using 350

V retarding voltage at an NEXAFS MCP detector to reject the lead NNV auger electrons (kinetic energy of about 250 eV). The samples measured at the FlexPES beamline were prepared and mounted in the same way as described in the main manuscript.

The MAPI spectrum shown in blue was measured on crystals synthesized with a slightly different method to those presented in the main manuscript by modification of a reported procedure by Zhang et al.[S1] Briefly, lead iodide (PbI_2, from Sigma-Aldrich) and methylammonium iodide (MAI, from Dyesol) were dissolved into γ-butyrolactone (GBL) to form a solution where the concentration of PbI_2 is 0.5 M and that of MAI is 1.5 M. Chloroform (≈680 µL/mL) was added to the aforementioned solution. The solution was filtered into a clean, 4 mL glass vial using a 0.45 µm filter. Chloroform was placed into another 4 mL glass vial. Both vials were placed inside a bigger vial, which was then capped and placed into an oil bath kept at 45 °C. This arrangement allowed the chloroform to slowly diffuse into the GBL solution. After two days of incubation, millimeter-scale MAPI crystals had formed. The crystals were collected and preserved in chlorobenzene, and blow-dried with nitrogen prior to use.

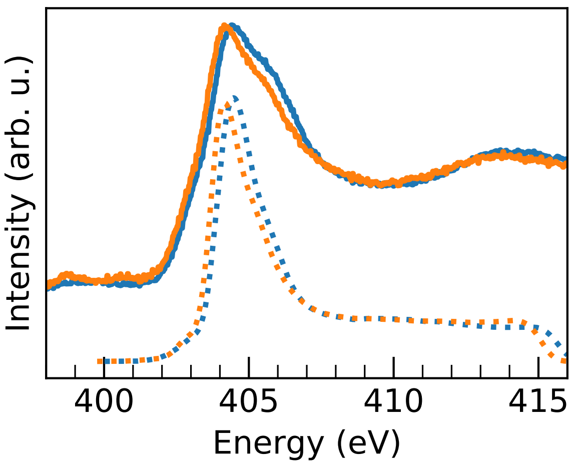

Figure S3: Comparison of experimental N K-edge XA spectra for MAPB (solid blue) and MAPI (solid orange) with the corresponding calculated spectra for MAPB (blue dash) and MAPI (orange dash) shifted by -2.86 eV. This shows the same data as Figure 2 with a longer energy scale to show the Pb N_5 edge as 415 eV. Note that the drop in the calculated spectra at 415 eV is due to the limited number of core-excited states.

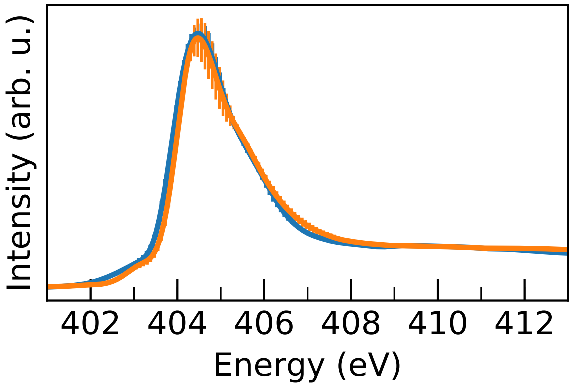

Figure S4: Comparison of average N K-edge spectra for sampled MAPB (blue) and MAPI (orange) geometries, with MAPI blueshifted by 0.31 eV to match the main peak with MAPB. Error bars represent the standard deviation between the different sampled spectra. This shows the good agreement of the two spectra when accounting for different absolute energies.

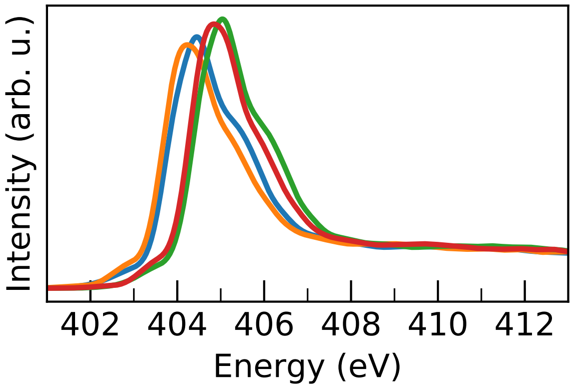

Figure S5: Comparison of 50 ps N K-edge spectra for MAPI and MAPB geometries, using the PBE (orange and blue) and BLYP (red and green) functionals. Pseudopotentials match the functional except for lead where the PBE functional was used for both. For PBE, peak intensities occur at 404.23 and 404.52 eV, respectively, while for BLYP they occur at 404.84 and 405.04 eV, respectively. This data shows that the relative differences between the two systems are similar with both functionals, in terms of the pre-edge, peak intensity differences, and post-edge shoulder.

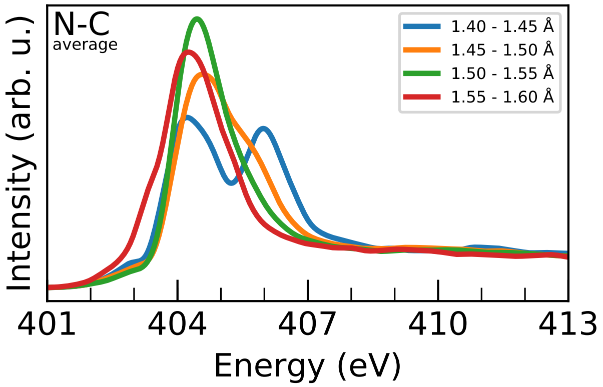

Figure S6: The block-averaged MAPB N 1s XA spectra ordered by N–C distance in 0.05 Å blocks from 1.40 to 1.60 Å. Explanation of the trends is given in the main text, and the block-averaging procedure is given in Figure 5.

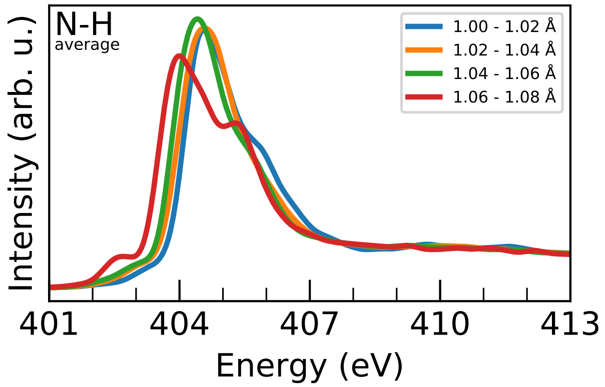

Figure S7: The block-averaged MAPB N 1*s* XAS spectra ordered by average N–H distance in 0.02 Å blocks from 1.00 to 1.12 Å. Explanation of the trends is given in the main text, and the block-averaging procedure is given in Figure 5.

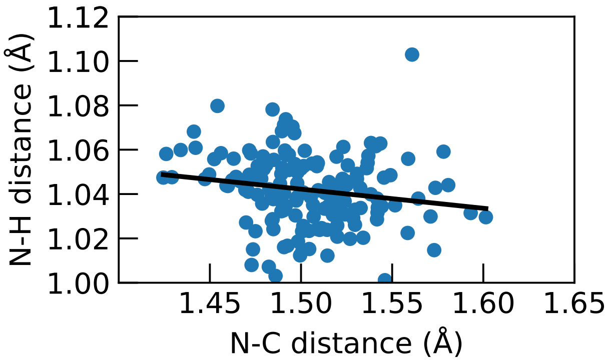

Figure S8: Scatter plot of N–C vs. N–H distance for the sampled geometries. The plot shows a slight inverse relationship between the two distances, in line with the results from Figures 7 and S7.

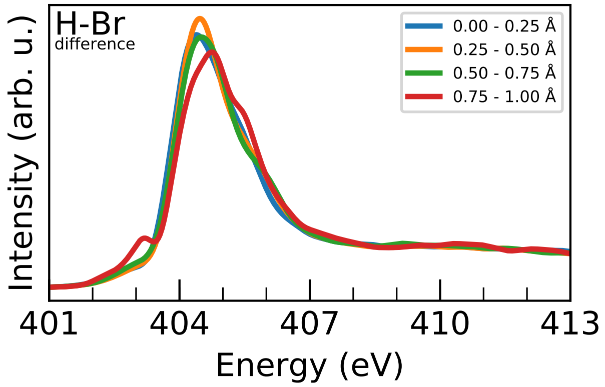

Figure S9: The block-averaged MAPB N 1*s* XAS spectra ordered by difference in shortest and longest nitrogen H$\cdots$Br distance in 0.25 Å blocks from 0 to 1 Å. Explanation of the trends is given in the main text, and the block-averaging procedure is given in Figure 5.

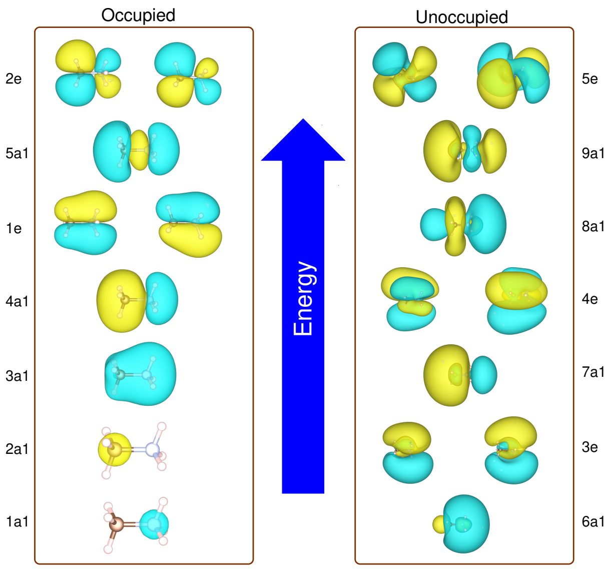

Figure S10: Molecular orbitals obtained from a ground state calculation of an isolated methylammonium (MA^+) ion.

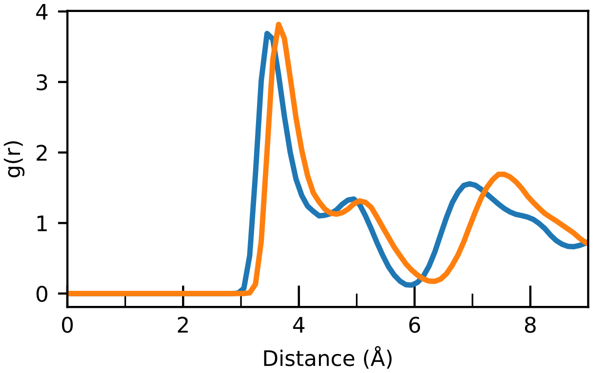

Figure S11: Radial distribution function (RDF) of N–Br (blue) and N–I (orange) distances during the 50 ps simulations of MAPB and MAPI.

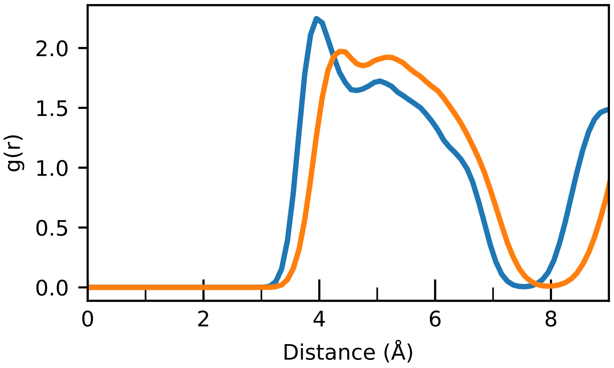

Figure S12: Radial distribution function (RDF) of H–Pb distances for nitrogen-attached hydrogens during the 50 ps simulations of MAPB (blue) and MAPI (orange).

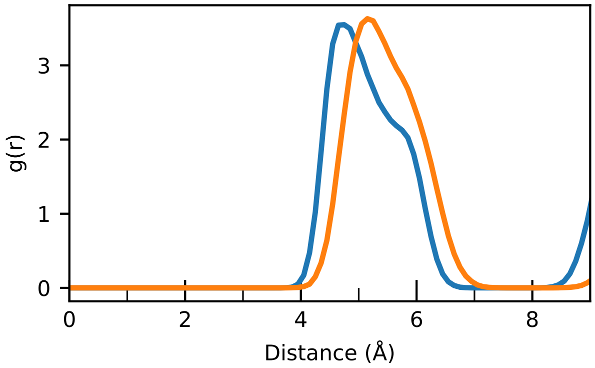

Figure S13: Radial distribution function (RDF) of N–Pb distances during the 50 ps simulations of MAPB (blue) and MAPI (orange).

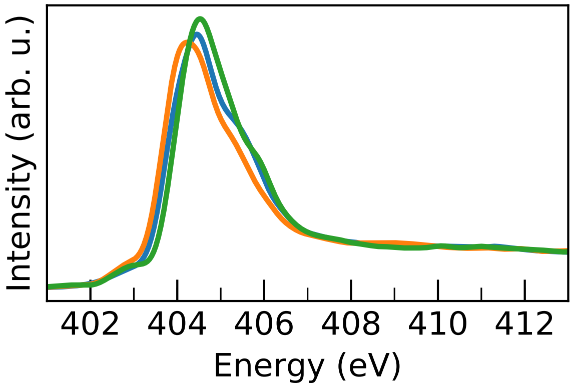

Figure S14: N 1s XAS spectrum for the 50 ps sample of tetragonal MAPI (orange) and cubic MAPB (blue) compared to the tetragonal MAPB sample after an additional 10 ps simulation at 200 K (green). The tetragonal MAPB reproduces a spectrum very similar to the cubic MAPB as opposed to the tetragonal MAPI, indicating the shift is an electronic effect rather than a geometric one.

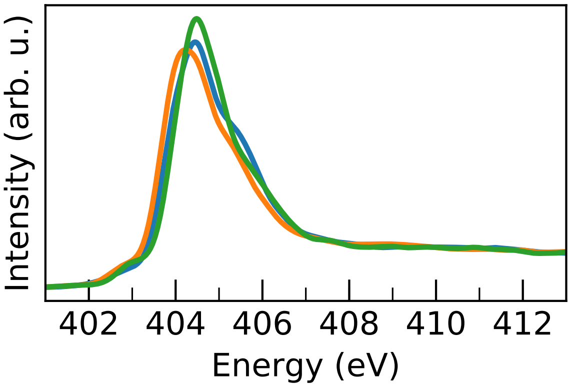

Figure S15: N 1s XAS spectrum for the 50 ps sample of tetragonal MAPI and cubic MAPB compared to the tetragonal MAPB sample after an additional 10 ps simulation at 300 K. The tetragonal MAPB reproduces a spectrum very similar to the cubic MAPB as opposed to the tetragonal MAPI, indicating the shift is an electronic effect rather than a geometric one.

References

(S1) Zhang, Y.; Huang, F.; Mi, Q. Preferential Facet Growth of Methylammonium Lead Halide Single Crystals Promoted by Halide Coordination. *Chemistry Letters* **2016**, *45*, 1030–1032.
